# Faecal microbiota and cytokine profiles of rural Cambodian infants linked to diet and diarrhoeal episodes

**DOI:** 10.1038/s41522-024-00562-0

**Published:** 2024-09-14

**Authors:** Matthew J. Dalby, Raymond Kiu, Iliana R. Serghiou, Asuka Miyazaki, Holly Acford-Palmer, Rathavy Tung, Shabhonam Caim, Sarah Phillips, Magdalena Kujawska, Mitsuaki Matsui, Azusa Iwamoto, Bunsreng Taking, Sharon E. Cox, Lindsay J. Hall

**Affiliations:** 1 Microbes, Infection and Microbiomes, College of Medicine and Health, University of Birmingham, Birmingham, B15 2TT UK; 2 Food, Microbiome & Health, Quadram Institute Bioscience, Norwich Research Park, Norwich, NR4 7UQ UK; 3 School of Tropical Medicine & Global Health, Nagasaki University, Nagasaki, Japan; 4 National Maternal and Child Health Centre, Ministry of Health, Phnom Penh, Cambodia; 5 Intestinal Microbiome, School of Life Sciences, ZIEL – Institute for Food & Health, Technical University of Munich, Freising, 80333 Germany; 6 Bureau of International Health Cooperation, National Centre for Global Health and Medicine, Tokyo, Japan; 7 Kampong Cham Provincial Health Department, Ministry of Health, Kampong Cham, Cambodia; 8 London School of Hygiene and Tropical Medicine, Keppel Street, London, WC1E 7HT UK; 9 Institute of Tropical Medicine, Nagasaki University, Nagasaki, Japan; 10 Norwich Medical School, University of East Anglia, Norwich Research Park, Norwich, NR4 7TJ UK

**Keywords:** Microbiome, Microbial genetics

## Abstract

The gut microbiota of infants in low- to middle-income countries is underrepresented in microbiome research. This study explored the faecal microbiota composition and faecal cytokine profiles in a cohort of infants in a rural province of Cambodia and investigated the impact of sample storage conditions and infant environment on microbiota composition. Faecal samples collected at three time points from 32 infants were analysed for microbiota composition using 16S rRNA amplicon sequencing and concentrations of faecal cytokines. Faecal bacterial isolates were subjected to whole genome sequencing and genomic analysis. We compared the effects of two sample collection methods due to the challenges of faecal sample collection in a rural location. Storage of faecal samples in a DNA preservation solution preserved *Bacteroides* abundance. Microbiota analysis of preserved samples showed that *Bifidobacterium* was the most abundant genus with *Bifidobacterium longum* the most abundant species, with higher abundance in breast-fed infants. Most infants had detectable pathogenic taxa, with *Shigella* and *Klebsiella* more abundant in infants with recent diarrhoeal illness. Neither antibiotics nor infant growth were associated with gut microbiota composition. Genomic analysis of isolates showed gene clusters encoding the ability to digest human milk oligosaccharides in *B. longum* and *B. breve* isolates. Antibiotic-resistant genes were present in both potentially pathogenic species and in *Bifidobacterium*. Faecal concentrations of Interlukin-1alpha and vascular endothelial growth factor were higher in breast-fed infants. This study provides insights into an underrepresented population of rural Cambodian infants, showing pathogen exposure and breastfeeding impact gut microbiota composition and faecal immune profiles.

## Introduction

Research on the gut microbiota has primarily focussed on populations in high-income countries (e.g. Europe and North America)^1^, and the problems and limitations this bias is introducing to microbiome research are gaining increasing attention^2^. Although more recent studies have increased the representation of populations in low- and low-middle-income countries (LMICs), infant studies lag behind those working on adult cohorts^3^. This is important for defining what a ‘healthy’ human gut microbiota^4^ is across individuals in LMICs^3^, and because optimal infant development is particularly important for infants from LMICs due to increased risk of (mal)nutrition and infection. While a small proportion of microbiota samples studied globally so far have come from South Asia, including India and Bangladesh^1^, research into the gut microbiota of people living in other countries in the region remains very limited. Moreover, due to the logistics of sample collection and storage in rural areas, research in LMICs has often focused on individuals in urban areas, neglecting key infant populations. Cambodia, in particular, has a scarcity of microbiota studies, and given that this country has such a high prevalence of infants classified as stunted/wasted and has amongst the highest infant mortality rates in Southeast Asia, with infections accounting for many of these deaths^5^, new studies targeting Cambodian infant populations are needed. Previous research on the gut-associated microbiota of people in Cambodia has been limited with studies focusing on particular bacterial pathogens, such as antimicrobial resistance in *Enterobacterales* in faecal samples from babies and children^6^, the epidemiology of antibiotic resistance in *Escherichia coli* and *Klebsiella pneumoniae* in children^7^, and effects of iron supplementation in Cambodian women^8^. However, an analysis of the composition of the gut microbiota as a whole in infants from Cambodia has not previously been published.

While the nutritional status of infants is improving in Cambodia, children under the age of 5 in rural areas still face challenges, with 22% suffering linear growth faltering or ‘stunting’^5^. This is also true for infants (<1 year) in rural Cambodia, who suffer from similar levels of growth stunting at 19.2%^9^, with a high proportion also lacking access to appropriate hygiene facilities. This is coupled with a high proportion reportedly experiencing symptoms of diarrhoea, fever or cough, and frequent use of unregulated antibiotic treatment to treat these childhood illnesses^10^. Malnutrition has previously been linked to differences in the gut microbiota of children from several different low-income countries^11,12^, although research into these associations is lacking for children from Cambodia.

The establishment of the gut microbiota during infancy is an important aspect of healthy infant development with roles in immune, metabolic, endocrine, and other host developmental pathways^13^. Vaginally delivered, breast-fed infants are characterised by their very high abundance of the bacterial genus *Bifidobacterium*. This keystone microbiota member orchestrates wider microbiome structuring and modulates host physiology through the breakdown of complex dietary components such as human milk oligosaccharides (HMOs) in breast milk and plant-based carbohydrates, leading to the production of key metabolites that facilitate immune system development and maturation and cognitive responses^14^. Previous research indicates that LMIC infant microbiomes are characterised by *Bifidobacterium* species and strains, although these are often different genetic variants when compared to those infants from high-income country settings^3,15^.

Infants born into resource-poor environments can have difficulties in establishing a healthy gut microbial ecosystem (Acosta et al, 2014)^16^ due to multiple factors such as birth and breast-feeding practices, antibiotic exposure, maternal microbiota, diet, socio-economic status, host genetics, agricultural dependence, urbanisation, access to clean water and hygiene practices^3,13^. Infant gut microbiomes of those in LMICs are reportedly higher in *Segatella* (previously *Prevotella*) species, associated with both positive and negative health outcomes, and may correlate with higher plant-based, fibre-rich diets^3^. The phylum Pseudomonadota (previously Proteobacteria), which includes a variety of pathogenic and opportunistic pathogens, is highly prevalent in microbiomes of those in LMICs^3^. These factors may negatively affect the composition of the early infant gut microbiota^13^ and may drive these observed microbiome differences across populations^3^.

In this study, we carried out an exploratory analysis of the gut microbiota in faecal samples collected from 32 infants that are part of the Nutrition for Health of Aka-chan (baby in Japanese) and Mamas’ cohort (NHAM) based in Cambodia, which included background data on infant diet, health, and living conditions. This provides an opportunity to explore the composition of the gut microbiota in rural Cambodian infants that has not previously been investigated. The samples from this cohort are unusual due to the rural location in which these infants live, with poor access to hygiene facilities, high prevalence of pathogen exposure, and frequent antibiotic usage. We analysed the effects of faecal sample storage on the composition of the microbiota of samples collected under suboptimal conditions. Then described the infant faecal microbiota composition and explored its associations with environmental factors, such as infant diet and growth. Individual faecal bacteria were cultured and isolated, and their genomes were sequenced to identify the presence of antibiotic resistance and HMO genes. Finally, the concentrations of faecal cytokines were measured to identify any associations with the gut microbiota. We aimed to explore the composition of the gut microbiota of these infants and its associations with their environment.

## Results

The longitudinal infant faecal samples analysed in this study were sourced from 32 infants representing a subset of infants that are part of the NHAM birth cohort based in the rural Kampong Cham province in southeast Cambodia, and ~60 km from the provincial capital Kampong Cham^9,10^ (Table 1). Samples were collected from infants living in a riverside community stretching along ~15.5 km of riverbank of the Mekong River in the communes of Khbop Ta Nguon, Preah Andoung, and Peam Koh Sna^9^. The infants were living in a rural area, with most families being small-scale farmers.

**Table 1:** Infant baseline characteristics

Variable	Infants
*n*	32
Age (days) (mean (SD))	214.9 (37.3)
Sex = Female (%)	15 (46.9)
Breast feeding = No (%)	9 (28.1)
Complementary feeding = No (%)	17 (53.1)
Feeding bottle use = Yes (%)	21 (65.6)
Low birth weight (<2.5 kg) (%)	
Normal	29 (90.6)
Unknown	2 (6.2)
Low	1 (3.1)
Growth = Stunted (%)	3 (9.4)
Length-for-age *z*-score (mean (SD))	−0.9 (0.8)
Underweight *z*-score (mean (SD))	−0.6 (0.8)
Weight-for-length (mean (SD))	0.0 (1.0)
Hand washing score (mean (SD))	5.2 (1.6)
Improved toilet facility = Yes (%)	22 (68.8)
Household toilet facility = Septic tank (%)	22 (68.8)
Number in household (mean (SD))	5.8 (2.2)
Antibiotic use (previous 7 days) = No (%)	28 (87.5)
Any recent illness (previous 7 days) = No (%)	11 (34.4)
Diarrhoeal illness (previous 7 days) = No (%)	26 (81.2)
Fever (previous 7 days) = No (%)	17 (53.1)
Cough (previous 7 days) = No (%)	20 (62.5)

### Sample storage conditions influence gut microbiota profiles

Sample collection and storage are challenging in areas without the facilities to immediately freeze faecal samples after collection. To help ensure better preservation of DNA in faecal samples, half of each faecal sample was immediately placed into a DNA preservation solution (DNAShield), with the other half remaining untreated. This later enabled the comparison of microbiota composition under different storage conditions. The faecal samples originated in a rural location with ~2 days between the sample being produced and final laboratory storage. Stool samples were collected by mothers or other caregivers and then kept in a relatively cooler place in the house or in cooler bags with ice until the field worker visited to collect them. After collection, the untreated half of each sample was transported in iceboxes and then initially frozen at −20 °C, and then later stored at −80 °C. Half of each sample stored in DNAShield was transported at ambient temperature and then stored refrigerated at 4 °C. Three samples from 32 infants were collected (a total of 96 samples), with the two halves of each sample split between the two storage conditions. Both frozen and DNAShield paired infant gut microbiota samples underwent 16S rRNA amplicon sequencing to analyse their bacterial composition.

**Fig. 1:**
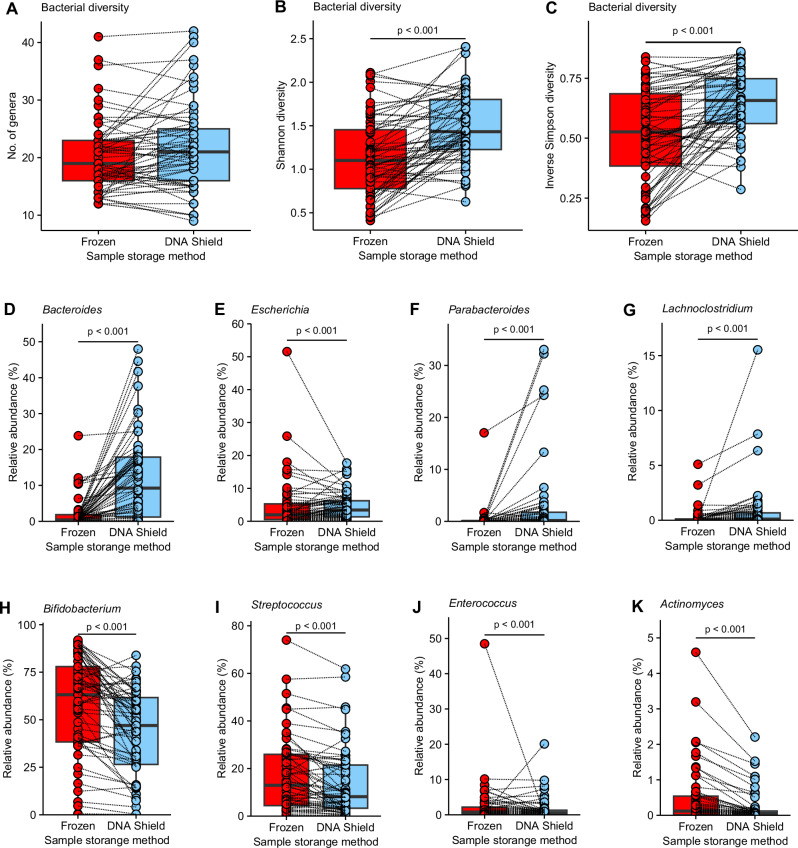
Differences in genus abundance between paired frozen samples and DNAShield stored samples. **A** The number of genera detected in each sample. **B** The Shannon diversity index of each sample. **C** The Inverse Simpson diversity index of each sample. Relative abundance between paired samples after different storage conditions of **D** *Bacteroides*, **E** *Escherichia*, **F** *Parabacteroides*, **G** *Lachnoclostridium*, **H** *Bifidobacterium*, **I** *Streptococcus*, **J** *Enterococcus* and **K** *Actinomyces*. Box plots show median and interquartile range with circles showing individual samples. Dotted lines link together paired Frozen and DNAShield samples from the same infant sample. Statistical significance between groups was tested using a Wilcoxon signed rank test with correction for multiple testing amongst bacteria genera using the false discovery rate method.

Out of the 96 paired samples, a total of 94 were successfully sequenced when stored in DNAShield, while only 70 were successfully sequenced from frozen samples (Supplementary Data 1). There were 69 pairs of samples with successful 16S rRNA amplicon sequencing from both the frozen and DNAShield portion of the same faecal sample. The resulting bacterial genus composition was compared between these 69 pairs of samples (Fig. 1). While the total number of bacterial genera detected was similar between frozen and DNAShield samples (Fig. 1A), microbiota diversity calculated using the Shannon index and Inverse Simpson index was higher in DNAShield samples than paired frozen samples (Fig. 1B, C). *Bacteroides*, *Escherichia, Parabacteroides, and Lachnoclostridium* were the four bacterial genera with the largest median difference in relative abundance between storage conditions that were higher in DNAShield samples compared to frozen samples (Fig. 1D–G; Supplementary Data 1). *Bacteroides* showed the largest difference in favour of DNAShield storage, with a median relative abundance of 0.4% in frozen samples and 8.6% in DNAShield samples (Fig. 1D; Supplementary Data 1). The relative abundance of *Bifidobacterium*, *Streptococcus*, *Enterococcus*, and *Actinomyces* were the genera with the largest difference and significantly higher abundance in favour of frozen samples (Fig. 1H–K; Supplementary Data 1). *Bifidobacterium* comprised a median relative abundance of 63% in frozen samples and 46% in DNAShield samples (Fig. 1H; Supplementary Data 1). Further analysis of bacterial microbiota composition in this study was performed using samples stored in DNAShield due to the larger number of samples with successful sequencing results and the higher abundance of individual bacterial genera surviving in DNAShield stored samples.

These results indicate that sample storage conditions can selectively alter the abundance of different genera in the analysis of bacterial composition with a significantly higher abundance of *Bacteroides* and *Parabacteroides* remaining when samples were stored in DNA preservation solution. This shows that DNA preservation solution can preserve genus abundance in final composition analysis even when samples are initially transported at ambient temperatures and then stored refrigerated and not frozen.

### Bacterial genus composition of the infant gut microbiota is influenced by breastfeeding and diarrhoeal illness

**Fig. 2:**
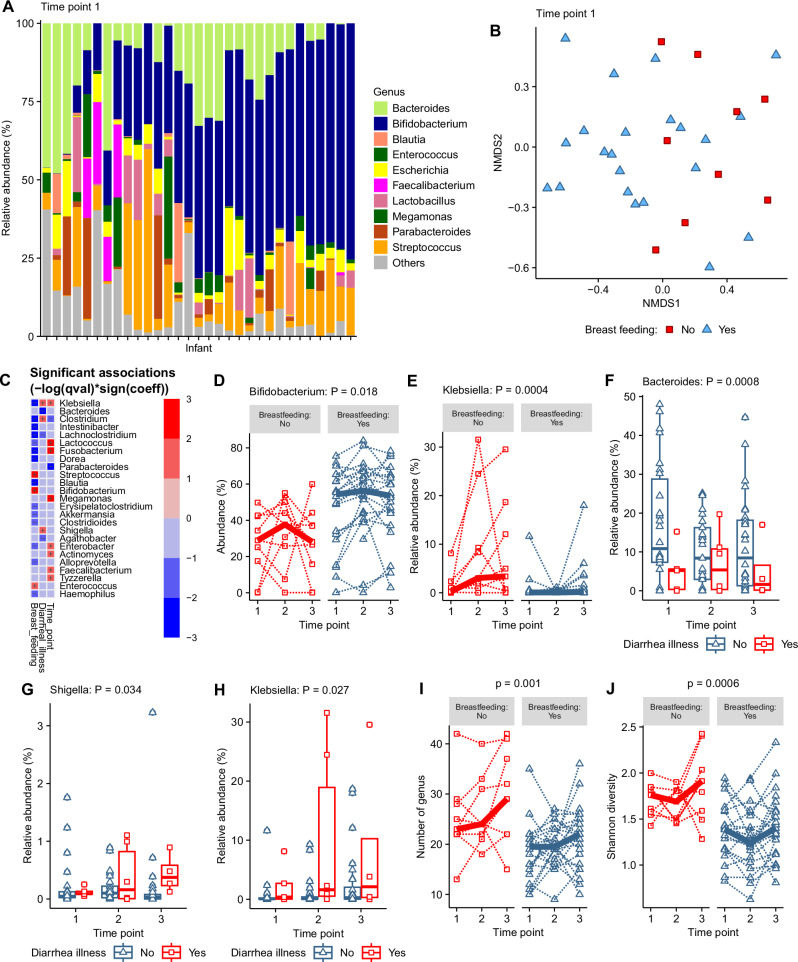
Genus composition and associations of genus composition with breast-feeding status and diarrhoeal illness. **A** The 10 most abundant bacterial genera across all samples are ordered on the *x*-axis by *Bifidobacterium* abundance in each infant. **B** Non-metric multidimensional scaling (NMDS) plot representing beta-diversity of infant genus microbiota at time point 1 clustered by similarity and coloured by breastfeeding status with arrows showing top 10 bacterial genera diving separation of samples. **C** Heat map showing significant associations determined by MaAsLin2 analysis between genus and breastfeeding, diarrhoeal illness and time point. **D** The relative abundance of the genus *Bifidobacterium* by breastfeeding status. **E** The relative abundance of the genus *Klebsiella* by breastfeeding status. **F** The relative abundance of the genus *Bacteroides* by diarrhoeal illness. **G** The relative abundance of the genus *Shigella* by diarrhoeal illness within the previous 7 days. **H** The relative abundance of the genus *Klebsiella* by diarrhoeal illness within the previous 7 days. **I** The number of genera by breast-feeding status. **J** The Shannon diversity index by breast-feeding status; Points on plots show individual infant data and dotted lines on spaghetti plots link individual infants across time points with the bold line showing the group median.

The gut microbiota of infants was dominated by *Bifidobacterium*, which comprised a median relative abundance of 48.7% (Fig. 2A). In addition, other genera, including *Bacteroides, Blautia, Enterococcus, Erysipelatoclostridium, Escherichia, Lactobacillus*, *Megamonas*, *Parabacteroides*, and *Streptococcus*, comprised the top 10 most abundant genera, and formed the majority of the gut microbiota in these infants (Fig. 2A).

An exploratory analysis was carried out using metadata available for the infants from which samples were collected. This included cross-sectional data collected at the start of the study before the collection of the faecal sample at time point 1 and additional information collected at each sample collection visit (Supplementary Data 2). The exploratory analyses used non-metric multidimensional scaling (NMDS) and sample beta-diversity to cluster the samples by the similarity of their overall microbiota composition at time point 1. Associations between the infant metadata and the beta-diversity of the gut microbiota were analysed using Envfit, and significant associations were illustrated on the NMDS plot (Fig. 2B). Only breast-feeding status was significantly associated with microbiota beta-diversity with clear separation in clusters between infants breast-fed and those infants not breast-fed (Fig. 2B).

To identify associations between the relative abundance of individual genera and infant metadata, an analysis using the MaAsLin2 package was carried out that included the samples from all three-time points, with the time point variable included as a random effect (Fig. 2C; Supplementary Data 3). When background metadata variables were analysed individually as a fixed effect, both breastfeeding status and recent diarrhoeal illness within the previous seven days showed significant associations with genus abundance, which was maintained when both variables were included together as a multivariable analysis (Fig. 2C). Genera with a differential abundance that was significantly associated with breast-feeding and/or diarrhoeal illness are shown as a heat map with positive and negative symbols indicating the direction of association (Fig. 2C). The genera positively associated with breast-feeding were *Bifidobacterium* and *Streptococcus* (Fig. 2C and D), whilst those with the strongest negative associations were *Klebsiella* and *Clostridium* (Fig. 2C and E). Breast-feeding status remained unchanged for individual infants across the three time points. At time point 1 the mean age of breast-fed infants was 207 days and non-breast fed infants 214 days. Including age as a variable in the analysis did not alter the associations with breastfeeding. Other measures of infant diet, the use of a feeding bottle or complementary feeding, were not associated with genus abundance. MaAsLin2 analysis also showed that recent diarrhoeal illness within the previous 7 days was associated with a lower relative abundance of *Bacteroides* (Fig. 2F) and a higher relative abundance of *Clostridium*, *Klebsiella* and *Shigella* (Fig. 2G, H). *Shigella* was detected in 78% of samples, *Klebsiella* in 82% and *Clostridium* in 65% indicating that these infants have a high prevalence of these potentially pathogenic genera in their gut microbiota (Supplementary Data 4). Breast-feeding was the only variable associated with differences in microbial diversity with the total number of the bacterial genus (Fig. 2I) and the Shannon diversity was lower in breast-fed infants than non-breast-fed infants (Fig. 2J).

Only three infants included in the analysis were classified as having stunted growth with a height-for-age *z*-score of less than 2 and this was not associated with microbiota composition. Tested as continuous measures, length-for-age *z*-score, underweight *z*-score, and weight-for-length *z*-score were also not associated with microbiota composition. However, this number of infants was too small for robust testing, preventing any strong conclusions being drawn. Measures of household hygiene facilities and the total number of people in the household were not associated with microbiota composition. Of particular note was the lack of an association between genus composition and recent antibiotic use within the previous 7 days (Supplementary Data 3).

These results indicate that breast-feeding was the most influential factor shaping the gut microbiota composition in favour of increased *Bifidobacterium* abundance. These results also show that recent diarrhoeal illness was associated with higher relative abundance of the potentially pathogenic genera *Clostridium*, *Klebsiella* and *Shigella*.

### Bacterial species composition of the infant gut microbiota is influenced by breast-feeding status

**Fig. 3:**
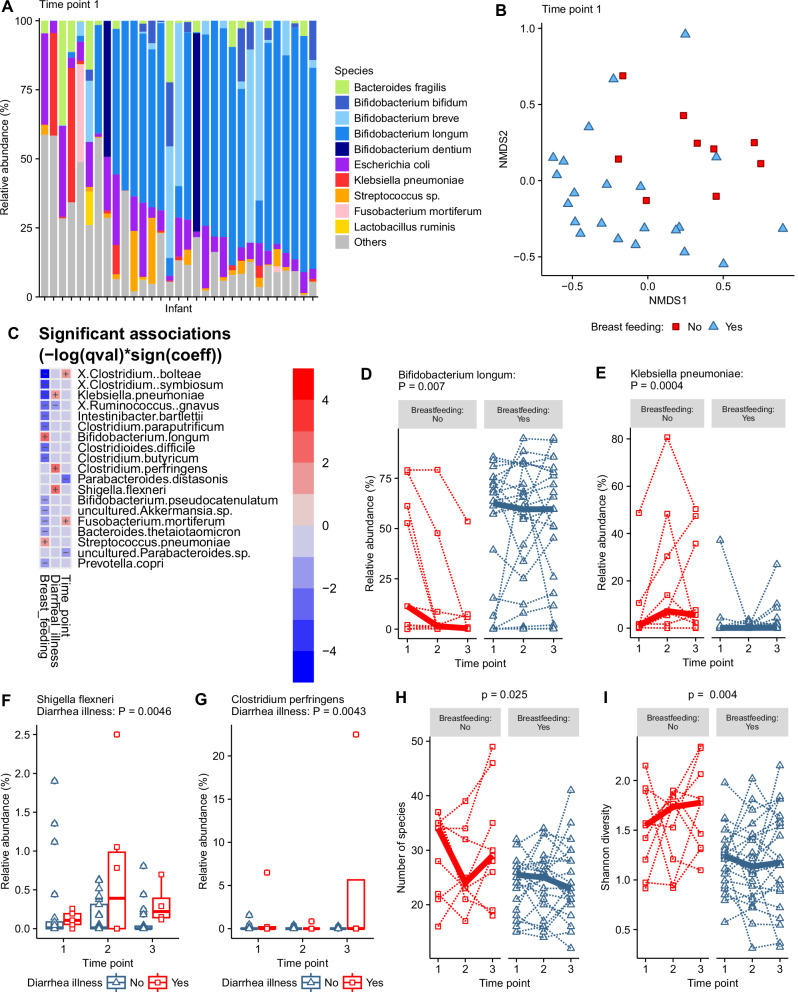
Species composition and association of species composition with breastfeeding and diarrhoeal illness. **A** The top 10 most abundant bacterial species across all samples at time point 1 ordered by total *Bifidobacterium* species abundance. **B** Non-metric multidimensional scaling (NMDS) plot representing beta-diversity of infant species microbiota at time point 1 clustered by similarity and coloured by breastfeeding status with arrows showing top five bacterial genus diving separation of samples. **C** Heat map showing significant associations determined by MaAsLin2 analysis between species and breast feeding, recent diarrhoeal illness and time point. **D** The relative abundance of the *Bifidobacterium longum* by breastfeeding status. **E** The relative abundance of *Klebsiella pneumoniae* by breastfeeding status. **F** The relative abundance of *Shigella flexneri* by recent diarrhoeal illness within the previous 7 days. **G** The relative abundance of *Clostridium perfringens* by recent diarrhoeal illness within the previous 7 days. **H** The number of species by breast-feeding status. **I** The Shannon diversity index by breast-feeding status, Points on plots show individual infant data and dotted lines on spaghetti plots link individual infants across time points with the bold line showing the group median.

The same analysis carried out on genus-level composition was carried out on the data at the species level with the limitation that species classifications using 16S rRNA amplicon data are less robust than genus (Supplementary Data 5). The gut microbiota at the species level was dominated by a single *Bifidobacterium* species classified as *Bifidobacterium longum* (Fig. 3A). Other species within the top 10 most abundant species at Time Point 1 included three other species of *Bifidobacterium* identified as *B. breve, B. bifidum*, and *B. dentium*, while *Escherichia coli*, *Enterococcus faecium*, *Klebsiella pneumoniae*, *Bacteroides fragilis* and an unclassified *Streptococcus* (Fig. 3A).

Repeating previous exploratory MaAsLin2 analysis of the species level 16S rRNA amplicon sequencing data identified the same association of breast-feeding status on microbiota composition with a stronger effect with species level data (Fig. 3C; Supplementary Data 6). Species with a relative abundance that was statistically significantly associated with breast-feeding and recent diarrhoeal illness are shown as a heat map with positive and negative symbols indicating the direction of association (Fig. 2C). *B. longum* showed the strongest positive association with breast-feeding, with consistently higher median relative abundance in breast-fed infants compared to those not breast-fed (Fig. 3D) while *Klebsiella pneumoniae* showed the strongest negative association with breast-feeding (Fig. 3E). The species *Shigella flexneri* (Fig. 3F) and *Clostridium perfringens* (Fig. 3G) were higher in samples taken after recent diarrhoeal illness. Breast-feeding was the only variable associated with differences in microbial diversity at the species level of classification. The total number of bacterial species (Fig. 3H) and the Shannon diversity was lower in breast-fed infants than in non-breast-fed infants (Fig. 3I). *S. flexneri* was detected in 49% of samples and *C. perfringens* detected in 24% of samples indicating that these infants have a high prevalence of these potentially pathogenic genera in their gut microbiota (Supplementary Data 7).

As described at the genus level, classification as stunted with a height-for-age *z*-score of <2 was not associated with gut microbiota species composition. Similarly, length-for-age *z*-score, underweight *z*-score, and weight-for-length z-score as continuous measures were also not associated with species composition. Hygiene measures of household toilet facilities and the total number of people in the household were again not associated with species composition. Also noted was the lack of an association between the gut microbiota composition and recent antibiotic use (Supplementary Data 6).

These species-level results support the finding that the breast-feeding status was a major influence on the gut microbiota composition and associated with the abundance of *B. longum*. Recent diarrhoeal illness was associated with higher abundance bacterial species including *S. flexneri* and *C. perfringens* that are known to be potential causes of diarrhoea.

### Infants harbour bacterial strains that encode diverse human milk oligosaccharide and antimicrobial resistance genes

**Fig. 4:**
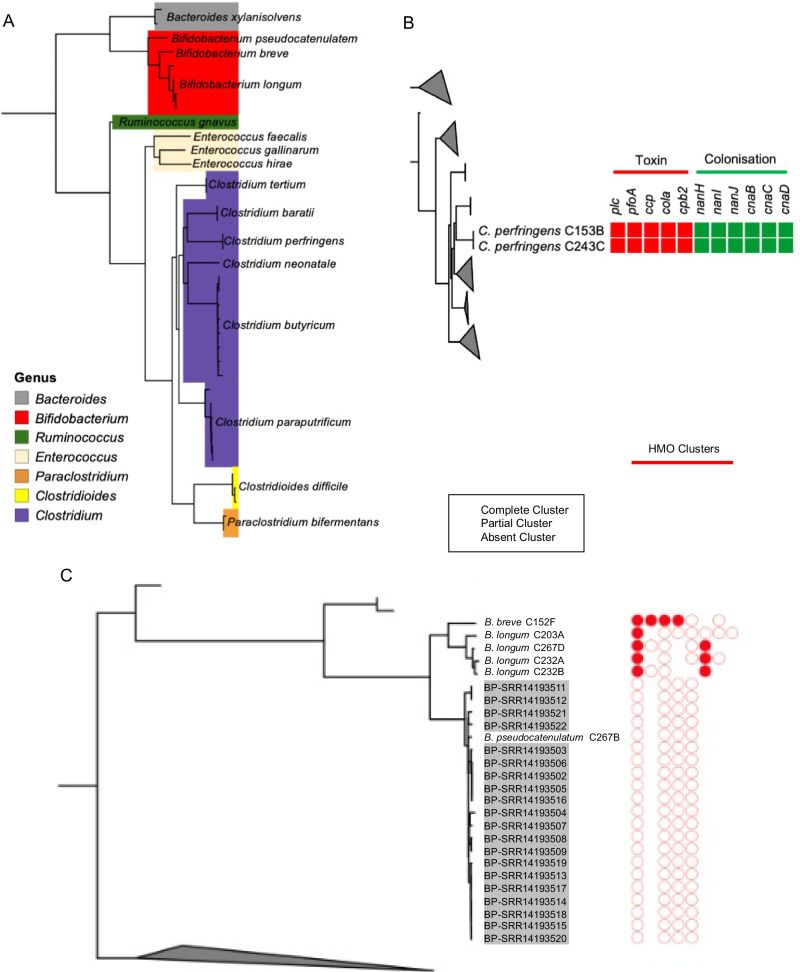
Genomic analysis of isolates for species determination, virulence genes, and human milk oligosaccharide (HMO) gene clusters. **A** Tree showing species and relatedness of whole genome-sequenced isolates from Cambodian infants. **B** Presence of toxin genes and colonisation factors in *Clostridium perfringens* isolates. **C** Presence of HMO gene clusters in *Bifidobacterium* isolates. Grey-shaded sample names indicate samples included for comparison from previous studies.

The frozen faecal samples that were not stored in DNA preservation solution were used to culture and isolated individual colonies for whole genome sequencing to gain further insight into the species of bacteria in the gut microbiota of these infants. Genomic analysis was carried out on the sequenced isolates to identify the species (and strains) and for the presence of virulence traits, antimicrobial resistance genes and HMO gene clusters. From the bacterial isolates genome sequenced, 2 were identified as *Bacteroides*, 6 as *Bifidobacterium*, 1 as *Ruminococcus*, 3 as *Enterococcus*, 21 as *Clostridium*, 3 as *Clostridioides*, and 2 as *Paraclostridium* (Fig. 4A).

Two *C. perfringens* strains were isolated and genome-sequenced. Both contained five toxin-producing genes and six colonisation factor genes that relate to the ability to cause infection and disease in the gut (Fig. 4B). This confirms the presence of *C. perfringens* that were identified using 16S rRNA amplicon sequencing and the presence of toxin and colonisation factor genes supports a potential role in its association with cases of infant diarrhoea.

*Bifidobacterium* isolates comprised three species, including *B. pseudocatenulatum*, *B. breve*, and *B. longum*. Comparing the four genome-sequenced bacterial isolates identified as *B. longum* using average nucleotide identity with a 98% cut-off, three were identified as *B. longum* subsp. *longum* and one as *B. longum* subsp. *suis* (Supplementary Data 8; Supplementary Fig. 1). Both *B. breve* and *B. longum* isolates contained complete HMO gene clusters, with *B. pseudocatenulatum* containing partial HMO gene clusters (Fig. 4C). The inclusion of *B. pseudocatenulatum* genomes isolated from infants in neighbouring Vietnam showed the same pattern of partial HMO gene clusters the Cambodian *B. pseudocatenulatum* isolate (Fig. 4C). This indicates the ability of the *B. breve* and *B. longum* present to digest the oligosaccharides present in human breast milk, with only *B. longum* showing a higher relative abundance in breast-fed infants compared to non-breast-fed (Fig. 3C, D).

**Fig. 5:**
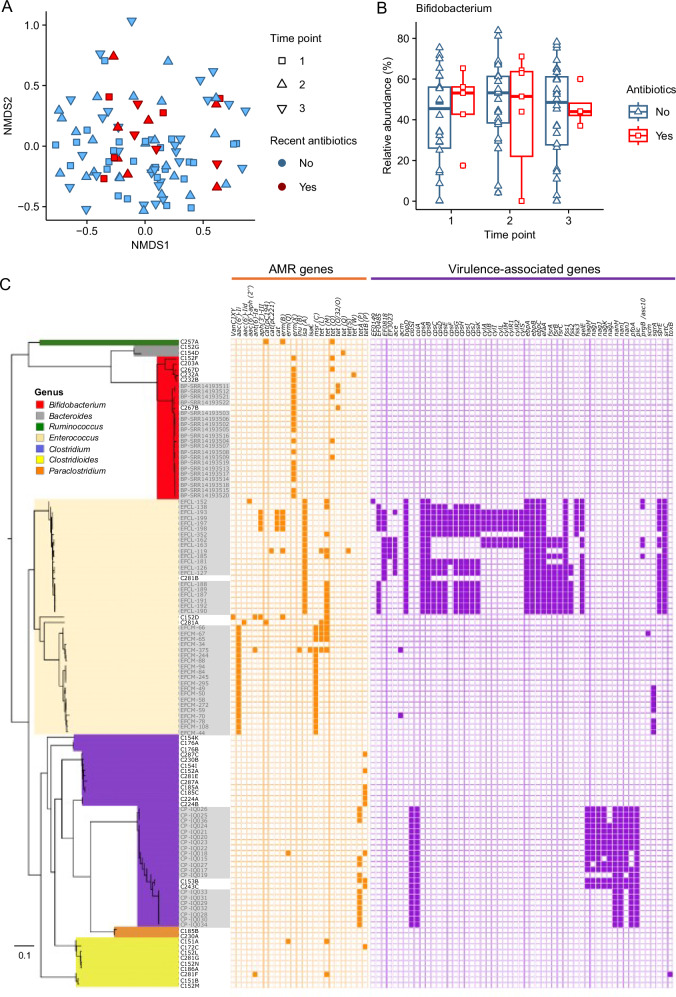
Compositional differences and *Bifidobacterium* abundance by recent antibiotic use and genomic analysis of isolates for antimicrobial resistance genes. **A** NMDS plot showing clustered faecal samples from all three time points coloured by recent antibiotic use. **B** *Bifidobacterium* genus abundance by antibiotic use. **C** Antimicrobial resistance genes and virulence-associated genes identified in the isolate genomes, plus public genomes. Grey-shaded sample names indicate samples included for comparison from previous studies.

Antibiotic use was not associated with compositional differences in the infant gut microbiota as shown using an NMDS plot showing the samples from all time points combined (Fig. 5A). Additionally, the relative abundance of *Bifidobacterium* did not show any difference in samples from infants receiving antibiotics (Fig. 5B). Genomic analysis of whole genome sequenced isolates showed the presence of antimicrobial resistance genes in the majority of strains isolated (Fig. 5C). Additional infant isolates of B. *pseudocatenulatum* originating from Vietnam and *E. faecium*, *Enterococcus faecalis*, and *C. perfringens* isolates originating from the United Kingdom some similar patterns of antibiotic resistance genes (Fig. 5C). Of particular note was the presence of the antimicrobial resistance genes *erm*(X), *tet*(O), *tet*(O/23/O), *tet*(Q), and *tet*(W) in the genomes of isolated *Bifidobacterium* species (Fig. 5C), which provide resistance to erythromycin (*erm*) and tetracycline (*tet*) respectively. Amongst this group of infants, the most commonly received antibiotic was amoxycillin, with individual instances of cefixime, and cefadroxile also received (Supplementary Data 2).

### Faecal cytokines profiles are associated with breastfeeding status

The early-life gut microbiota is known to play a key role in shaping gut and mucosal immune responses. A multi-plex assay, including a panel of 30 cytokines, was used to determine concentrations in the infant faecal samples (Supplementary Data 9).

**Fig. 6:**
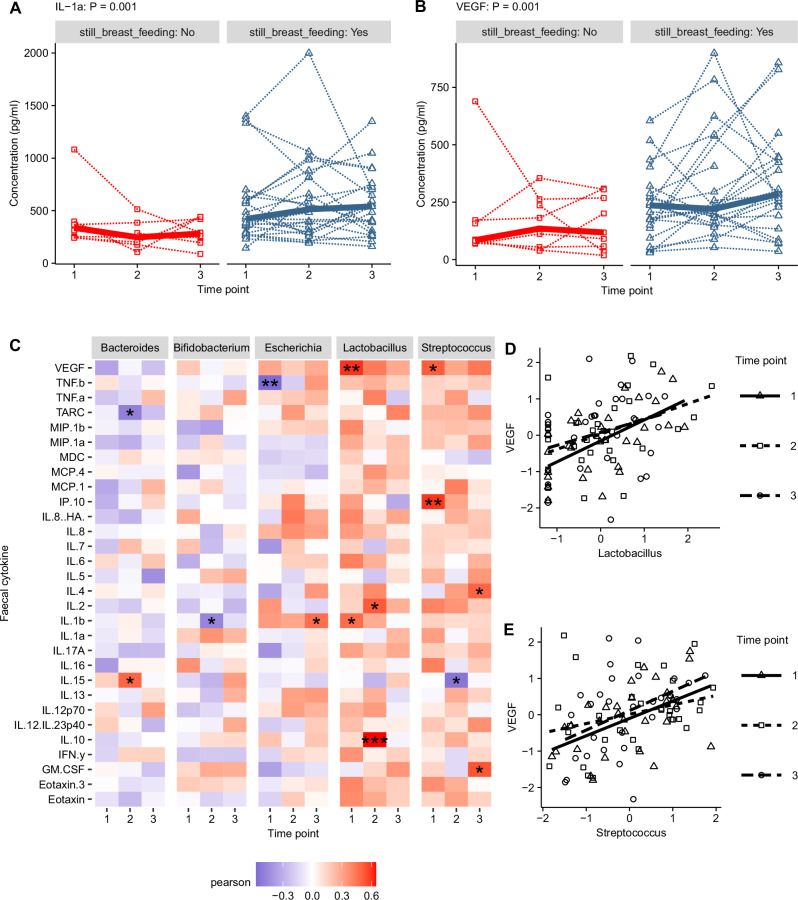
Infant faecal cytokines concentration and associations with breast feeding status. **A** Individual infant IL-1α concentration by breastfeeding status across time points. **B** Individual infant VEGF concentration by breastfeeding status across time points. **C** Pearson correlations between faecal cytokine concentrations and five highest abundance genus with variables in both datasets individually normalised using the bestNormalize package. **D** Scatter plot of VEGF and *Lactobacillus* correlations labelled for each time point. **E** Scatter plot of VEGF vs. *Streptococcus* correlations labelled for each time point.

A linear mixed model incorporating infant metadata variables as a fixed effect and the infant as a random effect was used as an exploratory analysis of associations between each cytokine and metadata variables in all samples across the three time points (Supplementary Data 10). The most robust and consistent association was identified between two cytokines and breast-feeding (Fig. 6A–B). The cytokines Interleukin-1-alpha (IL-1α) (Fig. 6A) and Vascular endothelial growth factor (VEGF) (Fig. 6B) were at significantly higher concentrations in breast-fed infants. Both cytokines showed substantial variation in concentration across time points within samples from the same infant. IL-1α (Fig. 6A) and VEGF (Fig. 6B) were also the two cytokines with the highest concentrations in the faecal samples across all infants.

To identify whether differences in major faecal cytokines by breastfeeding status were linked to differences seen in the microbiota composition a correlation analysis was carried out between faecal cytokine concentrations and the relative abundance of the 5 most abundant genus (Fig. 6C) and species (Supplementary Fig. 2) in the microbiota. Weakly consistent positive associations were seen between VEGF and *Lactobacillus* (Fig. 6D) and *Streptococcus* (Fig. 6E) across the three time points, significant at two time points after correction for multiple testing. There were no consistent correlations between the concentration of other cytokines and the relative abundance of individual bacterial genus or species, including *Bifidobacterium*.

## Discussion

This study represents the first time that the infant gut microbiota composition of a cohort of rural Cambodian infants has been analysed in detail. We show that a shortage of samples under rural conditions can radically reduce the abundance of certain genera within the samples. Infants had a gut microbiota dominated by the genus *Bifidobacterium*, shaped to a large degree by breastfeeding, which also impacts faecal cytokine profiles. Previous gut microbiota research in Cambodia has been limited to an investigation into antimicrobial resistance genes in Enterobacterales^6^, the epidemiology of antibiotic resistance in *E. coli* and *K. pneumoniae*^7^, and the effects of iron supplementation on individual genus abundance in women^8^. This present study goes further and examines in-depth genus and selected species and strain composition in Cambodian infants.

Our study results represent an unusual example of different sample storage conditions, as these samples were stored without immediate freezing. Overall, the effect of storage conditions on the composition of the microbiota was small, but selectively affected individual genera with a higher relative abundance of *Bacteroides* in particular was seen in samples stored in DNA preservation solution and a lower abundance of common species, including *Bifidobacterium* due to the proportional nature of the microbiota data. *Bacteroides* represents an important genus in the infant gut microbiota, transferred from the mother during delivery and has been found to be higher in vaginally delivered infants^17^, with some *Bacteroides* species having the ability to consume components of breast milk^18^. The relatively high proportion of *Bacteroides* in these infants and their almost disappearance from samples without DNA preservation solution shows the importance of a storage method that preserves this significant component of the infant microbiota for analysis.

Previous research has investigated the effects of storage conditions on microbiota analysis and reported alterations in *Bacteroides* abundance, although this has focused on storage conditions while frozen or the number of freeze–thaw cycles^19,20^. Room temperature storage of faecal samples for up to 72 h has been reported to reduce genus, including *Bacteroides*, in adult faecal samples over time^21^. While defrosting frozen samples for 3 h before analysis reduced *Bacteroides* abundance by half^22^. The duration of cool but unfrozen temperatures that samples in the present study were exposed to appear to have resulted in the particular low of *Bacteroides*. Other novel sample storage methods have been used for samples collected in difficult conditions. One study comparing rainforest hunter-gatherers and agriculturalist Bantu farmers in the Central African Republic used RNAlater or ethanol to preserve faecal samples for microbiota and metabolomic analysis, respectively^23^. Another unusually difficult situation collecting faecal samples from wild mountain gorillas and chimpanzees in Uganda found similar DNA preservation resulting from faecal storage in either RNAlater solution or dried using silica gel beads, although no microbiota analysis was carried out in this case^24^. However, the unpreserved samples in the present study were also important for culturing and isolating live bacteria for genomic sequencing, highlighting the importance of having samples stored without preservation when possible. There is an opportunity for future research to directly compare the effects of a greater range of storage conditions on downstream microbiota analysis to determine optimal methods for the collection of samples in suboptimal conditions.

The overall composition of the gut microbiota in these Cambodian infants was predominantly comprised of the genus *Bifidobacterium*. Comparative research indicates that a high abundance of *Bifidobacterium* is typical among infants in rural areas of less industrialised countries^25^ whereas it is generally lower in more westernised countries, such as the United States^26^, and also reported to be low in younger infants from China^27^. Other abundantly present genera in Cambodian infants were similar to those observed in other regional populations. For instance, infants from Bangladesh of a similar age exhibited the presence of *Lactobacillus*, *Streptococcus*, *Escherichia*, and *Klebsiella*^28^. One genus notably absent from this cohort of Cambodian infants is *Prevotella* (now reclassified under new genus names including *Segatella*), which has been observed in Bangladeshi infants at a similar age^28^, and found to be a dominant member of the gut microbiota in rural West Africa, yet rare in Italian children^29^. More recent studies have confirmed the high prevalence of *Segatella* in children from low-income countries such as The Gambia, Kenya, and Mali^30^, and specifically in Gambian infants from an early age similar to the Cambodian infants in the current study^31^. While *Segatella* abundance was lower in Bangladeshi children, it was still detectable in children of a similar age to this cohort of Cambodian infants^30^. In contrast, Cambodian infants exhibited the presence of *Bacteroides*, a genus typically associated with the gut microbiota of European and US inhabitants^29^. The dietary differences that may account for these discrepancies cannot be determined from this study alone, underscoring the need for further investigation into underrepresented populations. Whether the presence of *Bacteroides* and the absence of *Segatella* persist into adulthood remains unknown, as the composition of the adult gut microbiota in this region has yet to be explored.

*Bifidobacterium* was present at lower abundance in non-breast-fed infants and not breast-feeding was associated with a higher abundance of potentially pathogenic bacterial genera like *Klebsiella* and *Clostridium*. Although the number of infants not breast-fed was too low to draw strong conclusions, previous research has indicated that increased *Bifidobacterium* in the preterm infant gut can help reduce the proportions of potential pathogens^32^. This is linked to the ability of *Bifidobacterium* to metabolise HMOs in breast milk and reduce gut pH to make it a more inhospitable environment for the overgrowth of opportunistic pathogens. *B. longum* was the dominant species in this cohort of infants, and *B. longum* was the only *Bifidobacterium* species with higher abundance in breast-fed infants. The relatively low abundance of *B. pseudocatenulatum* in these infants may be due to their lack of complete HMO gene clusters. The matching pattern of partial HMO gene clusters *B. pseudocatenulatum* from Cambodia and Vietnam suggests a similar dietary niche for the species in infants in the region. The four *B. longum* isolates genome-sequenced all contained one or two HMO gene clusters indicating that they were potentially capable of metabolising the sugars present in breast milk. Interestingly, three of the *B. longum* isolates were confirmed to be *B. longum* subsp. *longum*, which do not typically dominate the gut microbiota of infants in high-income countries nor other LMICs with high rates of breastfeeding. It was recently reported that *B. longum* subsp. *infantis* was the dominant *Bifidobacterium* in 1–2-month-old infants from the Gambia and Bangladesh with *B. longum* subsp. *longum* only forming a minor proportion of the microbiota^15^. The isolation of *B. longum* subsp. *longum* from Cambodian samples may be due to sampling at an older age of around 7 months, with recent research from Bangladesh indicating that a transition occurs from *B. longum* subsp. *infantis* to *B. longum* subsp. *longum* with increasing infant age^28^. One *B. longum* isolate was identified as most closely matching *B. longum* subsp. *suis*, a subspecies originally isolated from pigs^33^ and found to be a dominant member of the piglet gut microbiota^34^. *Bifidobacterium* isolates corresponding to *B. longum* subsp. *suis* were recently identified in Bangladeshi infants^28^, which suggests that this may be an under-recognised member of the infant gut microbiota. From only a single isolate we cannot determine the proportion of *B. longum* subsp. *suis* within Cambodian samples, and this could represent the transient colonisation of this bacterium from close proximity to pigs or that this is a resident of the gut microbiota in this region. The presence of an HMO gene cluster capable of metabolising breast milk sugars indicates that this *B. longum* subsp. *suis* isolate may be capable of metabolising breast milk HMOs. The dominant abundance of the species *B. longum* in breast-fed infants underline the central effect of diet on the gut microbiota composition of these infants, overwhelming any other environmental factor in this small cohort.

Previous research has identified associations between restricted infant growth and stunting due to malnutrition and differences in the infant gut microbiota^12,35^. We did not find any associations between the gut microbiota and infant's growth. This was potentially due to the small cohort of infants involved, with only three out of the thirty infants included showing stunted growth and the relatively young age of the infants starting at an average of 7 months of age. Previous research has looked at older children^35^ or older infants, with a higher proportion of the infants suffering from stunting^12^. This indicates that in this small cohort of infants it is the presence or absence of breast milk having the largest effect, potentially overwhelming the ability to detect other environmental influences.

In Cambodia and many other LMICs, infants can be exposed to pathogens through the provision of contaminated drinking water in the first months of life (Poirot et al., 2018). Whilst we show that there are high proportions of beneficial bacteria in most samples, pathogenic taxa, including *Shigella*, *Escherichia*, and *Clostridium* genus were also detected in most infants. Although *S. flexneri* and *C. perfringens* abundance in the gut was associated with recent diarrhoea these bacterial species were also detectable in a large minority of infant samples indicating environmental exposure to these organisms and potential asymptomatic carriage.

Antibiotics can significantly alter the profile of early gut colonisation, resulting in reduced diversity^32^^,^^36^ and susceptibility to other infections^3^. Notably, in Cambodia, there is widespread, unregulated and inappropriate antibiotic use, and these selection pressures may further facilitate the dissemination of AMR genes^7^. An estimated one-third of the Cambodian population is <18 years old, and this group may be a significant reservoir for the spread of antimicrobial-resistant organisms^7^. Indeed, we observed multiple antibiotic-resistance genes in potentially pathogenic species, including those associated with diarrhoeal episodes. The pattern of antimicrobial resistance genes in *C. perfringens, E. faecium*, and *E. faecalis*, showed some similarities between Cambodian infant isolates and United Kingdom infant isolates and between *B. pseudocatenulatum* from Cambodian and Vietnamese infant isolates. This suggests geographically distant infant-derived bacterial genomes can share similar antibiotic-resistance genes. As these samples were from healthy infants, this potentially indicates a pattern of normal resistance in commensals in the gut. We also observed the presence of antimicrobial resistance genes in *Bifidobacterium*. Although this may seem unexpected, as *Bifidobacterium* is very rarely reported to be antibiotic resistant, a recent report from the neighbouring country of Vietnam indicates that *Bifidobacterium* isolated from healthy infants harbour antibiotic resistance genes^37^. We also found that Cambodian *Bifidobacterium* isolates contained resistance genes to the antibiotics erythromycin and tetracycline. This may link to the fact that this cohort of infants has previously been reported to be exposed to frequent and unregulated use of antibiotics^10^, thus potentially driving AMR dynamics in beneficial members of the infant gut microbiota. However, although amoxicillin was the most commonly used antibiotic in this cohort, we did not observe any beta-lactam resistance genes in the isolates. Previous studies have indicated that while it appears, there is high and unregulated use of antibiotics within the Cambodian population—they may be ineffective due to inappropriate storage, thus reducing their effectiveness. However, the widespread presence of resistance genes in other isolates may point towards active selection through exposure to antibiotics. The apparent maintenance of *Bifidobacterium* abundance despite antibiotic treatment may point to the presence of other novel resistance genes in these *Bifidobacterium*, which, if identified, may have future potential for combining effective probiotic therapy with antibiotic treatment.

Consistent differences in the faecal concentrations of the cytokines IL-1α and VEGF point to the potential effects of breastfeeding on the infant's gut mucosal immune system. VEGF has been reported to be secreted by the mammary glands, and breast milk contains high concentrations of free VEGF that can bind to receptors on the surface of cells in the infant intestine^38–40^. Reduced VEGF in preterm infants is suspected to be involved in necrotising enterocolitis, which is associated with the overgrowth of opportunistic pathogens, including *C. perfringens*^41,42^. While the role of VEGF originating from breast milk in term infants remains unexplored, these growth factors could exert growth-promoting and protective effects on the infant gut^38^. While the role of IL-1α in the infant's gut has not been explored, it has also been reported to be present in breast milk^43^; however, whether this has any effects on the infant's gut is currently unknown. Although we are unable to demonstrate a breast milk origin from these results, the presence of higher concentrations of these three faecal cytokines in breast-fed infants does suggest that they may be derived from breast milk, with a potential to influence infant development that warrants further investigation.

While there was little consistent association across time points between the gut microbiota at the genus level and faecal cytokines, a weak positive correlation was noted between *Lactobacillus* and *Streptococcus* and the faecal cytokine VEGF. As *Streptococcus* abundance was higher in breast-fed infants, this correlation is potentially linked to breastfeeding. As both microbiota composition and faecal cytokines are variable with time, it may not be possible to identify links between factors in a limited sample size of infants. Interestingly, despite being the genus most differentiated by breast-feeding status, there are no consistent correlations between *Bifidobacterium* IL-1α or VEGF. Indeed, many species and strains from this genus are known to positively influence the gut and systemic immune system—including *B. longum*, which is proposed to be in part mediated by metabolites produced as a result of HMO degradation, e.g. acetate^14,44^. However, further studies, including a larger number of infants, would need to be performed to explore microbial-immune interactions in more detail. In addition, the use of the frozen samples for faecal cytokine analysis may be limited as it is unknown how much storage conditions may impact the degradation of the cytokines tested.

The initial sample storage was pragmatic, as the samples were not collected with the intention of determining the effects of storage conditions on microbiota composition. A study specifically designed to investigate this question would include more detailed documentation of individual sample collection methods and compare various storage conditions. The available samples for microbiota analysis covered a short duration, with infants aged between an average of 201 to 235 days. These samples included infants who were either still breastfeeding or had stopped but did not cover periods before and after weaning. This allowed for a cross-sectional analysis of microbiota composition but limited the ability to assess how the infant microbiota developed over time. Future research could address this gap by collecting longitudinal samples from birth through childhood, providing a more comprehensive investigation of the maturation of the infant microbiota. Conducting such a study with infants in Cambodia would offer valuable insights and present an important opportunity for further research.

We show that this cohort of infants from rural Cambodia has a *Bifidobacterium*-dominated gut microbiota (including atypical species) that is primarily shaped by breastfeeding. This highlights the influence of breastfeeding in supporting a beneficial infant microbiota in rural conditions. As *Bacteroides* represents a common and important genus in the human gut the effects of sample storage are an important consideration when immediate freezing of samples is not possible. The lack of impact of antibiotic use may indicate either widespread antibiotic resistance or the use of ineffective antibiotics. The finding that faecal cytokines are shaped by breast-feeding and could potentially originate from breast milk is a finding with unknown but potentially interesting implications. This study adds significantly to the available data on the composition of infant gut microbiota communities in a previously neglected and difficult-to-sample geographical area of the world. Moreover, given that Cambodian profiles are somewhat different from other infant cohorts, including LMICs, studies such as these show the importance of looking at infants from underrepresented areas for defining what can constitute a normal infant microbiota.

## Methods

### Study design and ethics

Samples were collected from a sub-group of infants enroled in the NHAM cohort based in villages along the Mekong River in rural Kampong Cham province in Cambodia. This registered all infants born between April 2016 and March 2019 with the objective of investigating the causes of stunting. Full ethical approvals, including National Ethical Committee for Health Research in the Ministry of Health, Cambodia (016) and the Ethical Committee of the National Centre for Global Health and Medicine in Japan (NCGM-G-001870-01), and methods for the collection of detailed meta-data for infants included in this sub-study are as previously described^9,10^. Weight and length were measured to the nearest 0.1 kg and 1 mm by trained field workers at each home visit using a weighing scale (Seca 877, Seca, Hamburg, Germany) and a length board (Seca 417, Seca).

### Sample collection

Infant faecal samples were analysed from a subset of 32 infants out of 47 enroled in a detailed 3-month longitudinal follow-up study^9,10^ collected between March and July 2017. Mothers/primary care-givers and their infants were visited by study field workers with three samples collected and analysed from each infant for a total of 96 individual samples. The mean age of infants was 201 days (Standard deviation: 32) at Time Point 1, 216 days (Standard deviation: 30) at Time Point 2, and 236 days (Standard deviation: 30) at Time Point 3. The trained field worker gave instructions on how to collect and equipped the caregivers with the material for the child's stool collection prior to routine home visits. Once stool sample collection by mothers or caregivers was completed, the sample was kept in a relatively cooler place in the house or in cooler bags with ice until the field worker visited to collect them. The majority of the samples were collected by the field worker within 10 h of the time of child defecation reported by mothers (maximum: 47 h and minimum: 15 min among all samples). Each sample collected was divided into two and half stored in DNAShield and stored unaltered at ambient temperatures until delivered to laboratory. The other half of the sample was stored in iceboxes with refrigerant and transported to a −20 °C freezer on the same day, and once delivered was stored at −80 °C. The first sample was collected after an initial visit where cross-sectional metadata was collected. Subsequent samples were collected at approximately two-week intervals, along with further individual visit metadata. For recent diarrhoeal illness parent or caregiver was asked at the initial visit whether the child infant experienced diarrhoea within the past 7 days and since the previous visit at each subsequent sample collection. Underweight *z*-scores (weight-for-age), weight-for-length z-scores, length-for-age *z*-score were calculated previously^9^. A handwashing score was generated as a composite score combining the frequency and consistency of handwashing with soap. Improved toilet facility was defined as having access to a toilet flushing to a septic tank or pit latrine compared to unimproved having only access to a pit latrine or no toilet facility. Household toilet facility was defined as having a toilet within the home flushing to a septic tank.

### DNA extraction of preterm stool samples

FastDNA Spin Kit for Soil (MP) was used to extract DNA from preterm faeces following manufacturer instructions, with extended 3 min bead-beating. DNA concentration and quality were quantified using a Qubit® 2.0 fluorometer (Invitrogen).

### 16S rRNA amplicon sequencing of faecal samples

16S rRNA region (V1–V2) primers were used for library construction as described previously^32^. This set of primers allowed the amplification of one 16S rRNA gene sequencing library containing 96 different samples. PCR conditions used were: cycle of 94 °C 3 min and 25 cycles of 94 °C for 45 s, 55 °C for 15 s and 72 °C for 30 s. Sequencing of the 16S rRNA gene libraries was performed using the Illumina MiSeq platform with 300 bp paired-end reads.

### 16S rRNA amplicon data analysis

The raw sequence reads obtained was subjected to quality filtering using trim_galore v0.4.3. The resulting filtered sequence reads were then mapped against the 16S rRNA SILVA database (SILVA_132_SSURef_tax_silva) using BLASTn v2.2.25+ with a maximum e-value threshold of 10e-3. This mapping process was performed separately for both pairs of sequences^45^. After completing the BLASTn alignments, the output files were annotated using the paired-end protocol of MEGAN6, employing the default Lowest Common Ancestor (LCA) parameters^46,47^.

Further analysis was conducted using R version 4.2.2 (2022-10-31 ucrt) and RStudio (2023.06.0+421). To determine the number of reads required to adequately represent the microbiota in each sample, rarefaction curves were generated using the vegan package in R. For the analysis at the genus level, the sample with the smallest number of reads contained 37,306 classified reads, which was deemed an acceptable minimum. For the species-level analysis, the sample with the second lowest number of reads contained 9317 reads, which was considered an acceptable minimum. The 16S rRNA gene sequence data were then normalised by subsampling using the rarefy function in the phyloseq package, resulting in an even depth of 37,306 reads for the genus data and 9317 reads for the species data. One sample was excluded from the species data analysis as it only contained 3965 reads.

### Anaerobic bacterial culturing and isolation from infant faecal samples

Anaerobic bacterial culturing and isolation were performed on 17 Cambodian infant faecal samples (C151, C152, C153, C154, C172, C176, C185, C186, C203, C224, C230, C232, C243, C257, C267, C281, and C287) following^48–50^. Sample preparation was performed under anaerobic conditions. YCFA media and PBS were pre-reduced in an anaerobic cabinet at 37 °C for a minimum of 12 h before culturing. Two samples, weighing 100 mg each were prepared from each faecal sample. The first sample was homogenised in reduced PBS, serially diluted, and plated directly onto the YCFA agar. The second sample was ethanol treated using 70% ethanol under ambient aerobic conditions for 1 h to kill vegetative cells; the sample was then washed three times with reduced PBS to remove the ethanol, homogenised in the reduced PBS, serially diluted and plated onto YCFA + sodium taurocholate agar to stimulate spore germination. All agar plates were placed in the anaerobic cabinet at 37 °C for 48 h. Single colonies were picked and streaked onto pre-reduced YCFA media to isolate and purify the colonies; YCFA media was pre-reduced in the anaerobic cabinet at 37 °C for a minimum of 12 h before isolating colonies. Colonies from the non-ethanol-treated samples were streaked onto the reduced YCFA agar plates, and colonies from the ethanol-treated samples were streaked onto the reduced YCFA + Sodium taurocholate agar plates. Plates were then left to incubate in the anaerobic cabinet at 37 °C for 48–72 h. This was carried out a second time to make sure the isolates were pure. Falcon tubes containing reduced YCFA broth were inoculated with a single colony from the YCFA and YCFA + sodium taurocholate agar plates and left to incubate in the anaerobic cabinet at 37 °C for 48hs, with bacterial stocks prepared in 1.5 ml cryovials with 30% glycerol YCFA broth, and stored at -80˚C. Additionally, two infant faecal samples C203 and C232 were processed for targeted *Bifidobacterium* isolation. Briefly, serially diluted aliquots of faecal homogenates (10^−1^–10^−6^ in PBS) were plated onto MRS agar supplemented with 50 mg/l mupirocin and 0.5 g/l l-cysteine HCL and incubated in an anaerobic cabinet at 37 °C for 48 h. Colonies with desired morphology were randomly selected and re-streaked on MRS agar to purity. Pure cultures were prepared in 1.5 ml cryovials with 30% glycerol-reinforced clostridial medium (RCM) and stored at –80 °C.

### Whole genome sequencing (WGS)

DNA extractions were performed using the MPbio Fast DNA Spin Kit for soil and bead beating (but with an extended 3 min bead beating time). DNA quantification was carried out using the Qubit 2.0 fluorometer. DNA from pure bacterial cultures was sequenced at Wellcome Trust Sanger Institute using 96-plex Illumina HiSeq 2500 platform to generate 125 bp paired-end reads as described previously^51^.

### Genomic analysis

Paired-end raw sequence reads (fastq) were quality-filtered (-q 20) via fastp v0.20.0^52^ prior to de novo genome assembly using SPAdes v3.14.1^53^ at default parameters. Next, contigs smaller than 500 bp were discarded in each genome assembly. All genome assemblies were subjected to contamination check via CheckM v1.1.3^54^, contaminated (contamination > 5%) and/or incomplete (completeness < 90%) genome assemblies were excluded from further analyses (*n* = 5). All genomes were identified taxonomically using gtdb-tk v1.5.1^55^, using ANI cut-off 95% against type strains of each species.

These high-quality genome assemblies (*n* = 71) were then dereplicated at ANI 99.9% (cut-off for strain level) using dRep v3.2.2^56^ prior to generation of a distance tree using Mashtree v1.2.0^57,58^ on default parameters. Additional children/infant-associated reference bacterial genomes (*n* = 80) from recent studies (retrieved from NCBI) were added for comparison purposes—*Enterococcus faecium*, *Enterococcus faecalis*, *Bifidobacterium pseudocatenulatum* and *C. perfringens* (Supp X)^37,41,58^. Genome reads (reference genomes) downloaded from SRA (Sequence Read Archive) were subjected to quality filtering (-q 20) by fastp v0.20.0 followed by genome assembly via unicycler v0.50.0 prior to further gene screening analysis. Mash-distance tree was annotated in iTOL v6.5.8^59^.

These genomes were also subjected to sequence search analysis via ABRicate v1.0.1^60^ based on ResFinder v4.0 database^61^ (--minid=95 and --mincov=90) to determine antimicrobial resistance (AMR) profiles, and VFDB database^62^ (--minid=90 and –mincov=90) to determine virulence profiles. Furthermore, HMO clusters were detected using an in-house HMO sequence database via ARBricate at –minid=70^63^. In addition, *C. perfringens* virulence profiles were predicted via database TOXIper v1.1 using ABRicate at –minid=90^64^.

For *Bifidobacterium longum* subspecies analysis, *B. longum* strains were compared with four subspecies type strains, namely, subspecies *infantis*, subspecies *longum*, subspecies *suillum* and subspecies *suis*, to generate a core gene alignment via Panaroo v1.2.8^65^, resulting in 1212 core genes, which core-gene alignment constructed by MAFFT^66^ were SNP-extracted (via SNP-sites v2.3.3)^67^ and subsequently used to generate a phylogenetic tree via IQ-TREE v2.0.5^68^ with ultra-fast bootstrap replicates -B 1000 and automatic evolutionary model selection option -m TEST (best fit model was determined to be GTR+F+ASC+G4). Subspecies identification was performed using fastANI v1.3^69^ at cut-off 98%.

### Faecal cytokine measurement

Naïve (i.e. samples not stored in DNASheild, which would interfere with cytokine analysis) faecal samples were homogenised with PBS using a FastPrep® Bead Beater (4.0 m/s, 3 min), centrifuged (14,000 rpm, 15 min) and 25 μl of supernatant was used for the assay. Samples were analysed using MULTI-SPOT™ plates, MESO Quickplex SQ120 and discovery workbench software according to the manufacturer’s protocol. Pre-coated immunoassays V-PLEX Proinflammatory Panel 1 (human) and V-PLEX Cytokine Panel 1 (human) were used to detect a set of 20 different cytokines: IFNγ, IL-1β, IL-2, IL-4, IL-6, IL-8, IL-10, IL-12p70, IL-13, TNFα, GMCSF, IL-1α, IL-5, 273 IL-7, IL-12p40, IL-15, IL-16, IL-17A, TNFβ, and VEGF-A.

### Statistical analysis

Statistical analysis was conducted using R version 4.2.2 (2022-10-31 ucrt) and RStudio (2023.06.0+421). To examine differences in the relative abundance of genera among the 69 infant faecal samples, which had 16S rRNA sequence data obtained from both samples stored in DNAShield solution and Frozen, a Wilcoxon rank sum test was performed in R. This test was chosen due to the non-parametric nature of the majority of the genus abundance data. To account for multiple testing, a Benjamini and Hochberg false discovery rate (FDR) method was applied, and a *p*-value of <0.05 was considered statistically significant for adjusted *p*-values.

The overall composition of the 16S rRNA gene sequence data was analysed using NMDS (Non-metric multidimensional scaling). The NMDS plots were generated in R Studio using the vegan package version 2.6-4, employing a Bray-Curtis dissimilarity calculation. To determine the statistical significance of differences in microbial community structure based on infant metadata variables, permutational MANOVA was conducted using the Adonis function from the vegan R package version 2.6-4.

Microbial diversity at both the genus and species taxa levels was calculated using the vegan package version 2.6-4. The number of genera or species was determined for each sample, and the Shannon diversity index was calculated accordingly. Linear mixed-effect models, utilising the “lmer” function in R, were employed to test the associations between the relative abundance of genera or species and metadata variables. The number of genera or species, as well as the Shannon diversity index, were assessed against each individual metadata variable, with Time Point included as a fixed effect and Patient ID included as a random effect in the model.

To test for a differential abundance of genera and species in relation to available background metadata variables for the infant samples, the Maaslin2 package version 1.12.0 (Microbiome Multivariable Associations with Linear Models)^70^ was utilised. The Maaslin2 analysis was performed with default parameters, including Benjamini-Hochberg FDR multiple testing correction and a significance threshold of 0.25. No minimum prevalence or abundance cut-offs were applied, and the data input was previously normalised. Each metadata variable was individually tested, incorporating Time Point as a Fixed Effect and Patient ID as a random effect grouping variable in a mixed-effect model. Positive associations were identified individually with Breast Feeding and Diarrhoeal Illness and these two variables where tested together in the same model as Fixed Effects with Time Point and with Patient ID included as a Random Effect in the final analysis.

Associations between faecal cytokine concentrations and metadata variables were tested using the MaAsLin2 package version 1.12.0^70^, differences in concentration against background metadata variables available for the infant samples. For faecal cytokine analysis, MaAsLin2 was run with default parameters, including multiple testing correction using Benjamini–Hochberg FDR with a significance threshold of 0.25, except no minimum prevalence or abundance cut-offs were applied, normalised methods used were “CLR” and the transformation “LOG”. Each metadata variable was tested individually with time point as a fixed effect and with patient ID included as a random effect grouping variable to run a mixed effect model.

Correlations between faecal cytokine concentration and the relative abundance of genus and species were tested using a Pearson correlation with correction for multiple testing using Benjamini and Hochberg FDR correcting between both cytokines and time points. Both cytokine concentration and genus/species abundance were first normalised using the bestNormalize package version 9.1.1 in R.

### Reporting summary

Further information on research design is available in the Nature Research Reporting Summary linked to this article.

## Supplementary information

Combined Supplementary Files

Supplementary File 1

Supplementary File 2

Supplementary File 3

Supplementary File 4

Supplementary File 5

Supplementary File 6

Supplementary File 7

Supplementary File 8

Supplementary File 9

Supplementary File 10

Reporting Summary

## Data Availability

Bacterial pure genome assemblies are now deposited at NCBI GenBank under project accession PRJNA958097, while 16S rRNA amplicon sequencing raw reads are deposited in NCBI SRA under project PRJNA958098.

## Code availability

Bacterial pure genome assemblies are now deposited at NCBI GenBank under project accession PRJNA958097, while 16S rRNA amplicon sequencing raw reads are deposited in NCBI SRA under project PRJNA958098.
