# Supplementary material for: Faecal microbiota and cytokine profiles of rural Cambodian infants linked to diet and diarrhoeal episodes
Source: NPJ Biofilms Microbiomes. 2024 Sep 14;10:85. doi: 10.1038/s41522-024-00562-0 (PMC11401897; doi:10.1038/s41522-024-00562-0)
Supplement: Supplementary file 1 — Combined Supplementary Files [file 41522_2024_562_MOESM1_ESM.pdf]

Supplementary Data

Supplementary Data 1. Genus differences between DNAShield and Frozen storage conditions.

Supplementary Data 2. Metadata and Genus Sequence Data

Supplementary Data 3. Genus data Maaslin2 analysis results.

Supplementary Data 4. Genus prevalence in samples.

Supplementary Data 5. Combined sample metadata and species sequencing data for all samples.

Supplementary Data 6. Species data Maaslin2 analysis results.

Supplementary Data 7. Species prevalence in samples.

Supplementary Data 8. Pairwise Average Nucleotide Identity (ANI)

Supplementary Data 9. Metadata and Faecal Cytokine Concentrations.

Supplementary Data 10. MaAsLin2 Cytokine Results

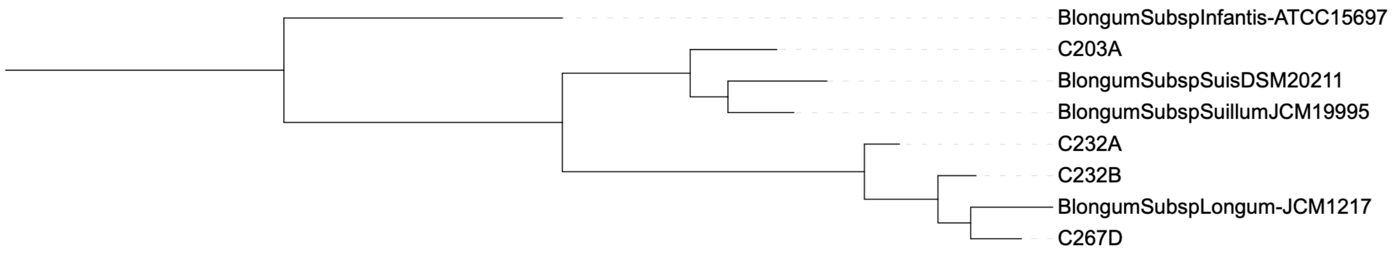

Supplementary Figure 1. The subspecies of Bifidobacterium longum isolates determined using average nucleotide identity with a 98% cut-off.

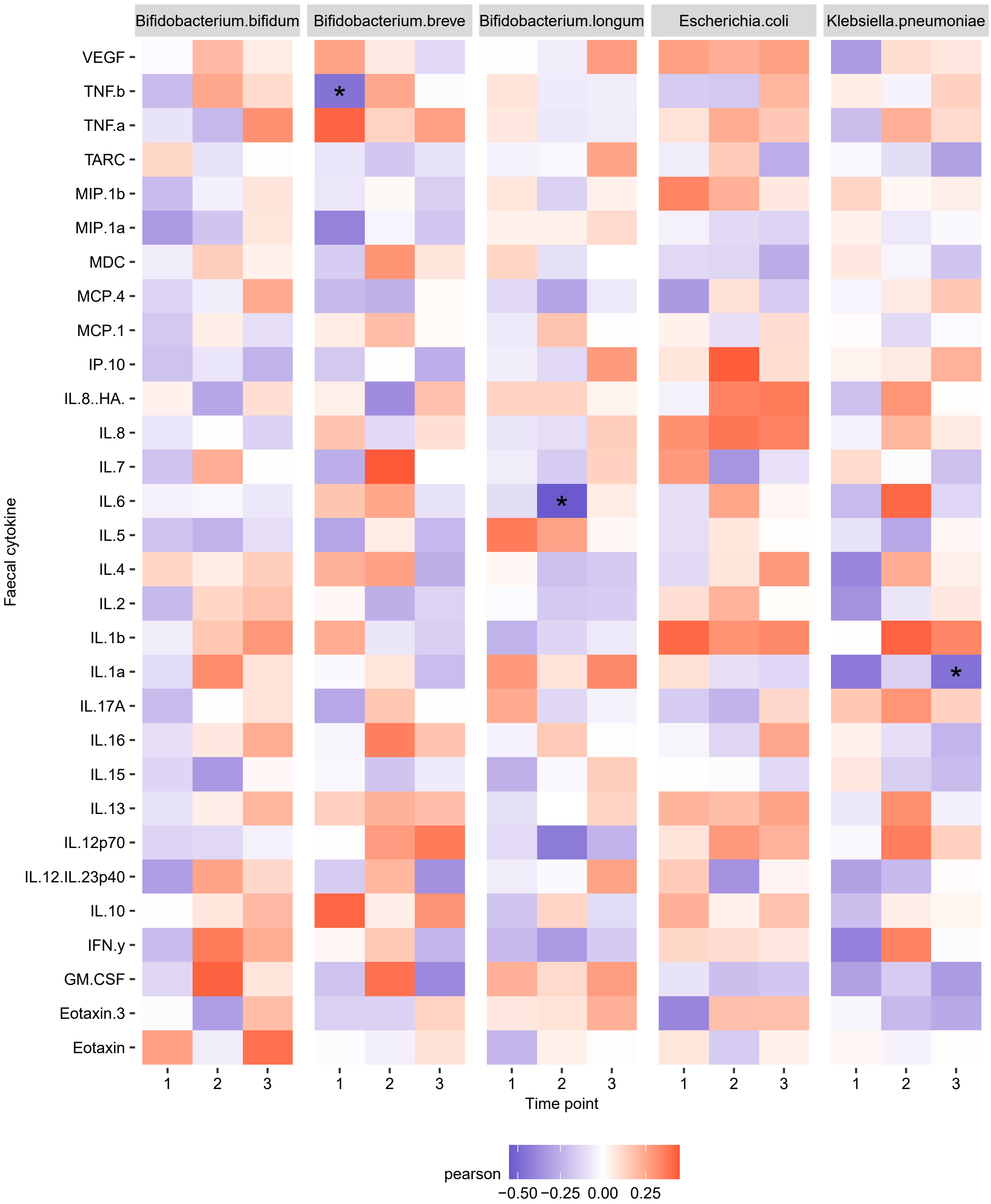

Supplementary Figure 2. Correlations between cytokines and Genus or Species relative abundance.
